# High-temperature stress response in the liver of *Trachinotus ovatus*: a cascade of oxidative stress, immunity, and bile acid metabolic reprogramming

**DOI:** 10.1007/s44154-026-00344-y

**Published:** 2026-10-19

**Authors:** Ruijie Zhu, Meng Xiao, Xiaohua Huang, Dianchang Zhang, Falin Zhou, Yukai Yang, Huayang Guo, Yafei Duan

**Affiliations:** 1 State Key Laboratory of Mariculture Biobreeding and Sustainable Goods, Key Laboratory of South China Sea Fishery Resources Exploitation & Utilization, Ministry of Agriculture and Rural Affairs, South China Sea Fisheries Research Institute, Chinese Academy of Fishery Sciences, Guangzhou, 510300 PR China; 2 College of Fisheries and Life Science, Shanghai Ocean University, Shanghai, 201306 China

**Keywords:** High-temperature stress, *Trachinotus ovatus*, Oxidative stress, Physiological response, Bile acid metabolism

## Abstract

Fish exhibit a high degree of susceptibility to shifts in thermal conditions within the water. The liver is central to metabolic homeostasis and immune defense, and its physiological stability is critical for fish health. The present study evaluated the hepatic physiological responses of *Trachinotus ovatus* following a 7-day exposure to high-temperature (HT) stress, including alterations in histology, oxidative stress, iron homeostasis, inflammation, immunity, apoptosis, and bile acid (BA) metabolism. HT stress induced alterations in liver histology and disrupted redox homeostasis, accompanied by enhanced Nrf2-mediated antioxidant signaling and changes in antioxidant defense enzyme activities and peroxide levels. Iron homeostasis and lipid peroxide defense exhibited complex alterations, including Fe levels and *NCOA4* expression were increased, accompanied by compensatory upregulation of iron-homeostasis genes (*FTH1*, *FPN1*, and *NFS1*) and antioxidant defense genes (*GPx4*, *SLC7A11*, and *p53*). However, reduced lipid peroxidation products (LPO and MDA) suggested that these changes reflected regulatory adaptation rather than the execution of ferroptosis. HT stress induced hepatic inflammatory and immune responses, characterized by activation of pro-inflammatory signaling, dysregulated anti-inflammatory regulation, downregulation of *IL-8* expression, and downregulation of the immunoglobulin genes *IgM* and *IgT*. Apoptosis-related genes showed divergent expression patterns, with *CHOP*, *Casp-9*, and *Casp-3* being upregulated, whereas *CytC* was downregulated. Furthermore, BA metabolism was affected, as shown by reductions in total BA pool size and free BA levels, along with the upregulated expression of BA metabolism-related genes (*FXR*, *SULT1*, and *UGT8*). Three metabolites, cholic acid, chenodeoxycholic acid, and 7-ketolithocholic acid were identified as potential biomarkers. Collectively, these findings suggest that HT stress impaired hepatic physiological homeostasis in *T. ovatus*, accompanied by tissue architecture disruption, redox imbalance, inflammatory responses, immune suppression, BA metabolic remodeling, and coordinated changes in iron homeostasis and apoptosis-related regulatory pathways.

**Supplementary Information:**

The online version contains supplementary material available at https://doi.org/10.1007/s44154-026-00344-y.

## Introduction

*Trachinotus ovatus* is an important aquaculture species with significant economic and ecological value (Wang et al. 2024). Rapid growth and high flesh quality have driven a marked expansion of *T. ovatus* production, and by 2024 the species had become one of China's principal mariculture fishes (Bureau of Fisheries of the Ministry of Agriculture 2025). However, frequent disease outbreaks have posed significant challenges to the stability of *T. ovatus* production, with environmental stress being a major contributing factor. In recent years, summer seawater temperatures have continuously increased, while episodes of extreme heat have become more frequent, with aquaculture environments in southern China reaching nearly 34 °C (Ma et al. 2021a; Yao et al. 2025; Yao and Wang 2021). Because fish are ectothermic, variations in environmental temperature can substantially alter their physiological functions (Alfonso et al. 2021). For *T. ovatus*, the temperature range of approximately 22–28 °C has been identified as optimal for growth (Li et al. 2023a), whereas exposure to temperatures above 32 °C adversely affects its growth, development, hatching success, and survival (Li et al. 2023a; Han et al. 2020). Moreover, high-temperature (HT) stress may increase the susceptibility of *T. ovatus* to bacterial and parasitic infections, thereby compromising aquaculture performance (Huang et al. 2024; Yin et al. 2023; Guo et al. 2023a). Although the critical thermal maximum (CTmax) of juvenile *T. ovatus* has not yet been established, 34 °C may represent a sublethal HT condition that can occur in aquaculture environments and induce physiological stress, potentially increasing its disease susceptibility. Accordingly, clarifying how *T. ovatus* responds physiologically to sublethal HT stress is important for improving its environmental adaptability and mitigating farming risks during the hot season.

Elevated temperature is a major environmental stressor that can substantially influence fish physiology. Under HT stress, reactive oxygen species (ROS) may accumulate beyond normal levels, disturbing cellular redox equilibrium and triggering antioxidant defense responses (Guo et al. 2023b). However, excessive ROS generation relative to antioxidant capacity may cause prolonged oxidative stress, thereby accelerating lipid peroxidation (LPO) and inducing cellular injury (Long et al. 2023b). ROS are not only mediators of oxidative injury but also signaling intermediates that influence inflammatory, immune, and apoptotic pathways. For instance, HT stress has been reported to promote hepatic inflammation, impair humoral immunity, and induce apoptosis across different fish taxa (Liu et al. 2022a; Lin et al. 2024; Xv et al. 2024). Therefore, redox disturbance represents a prominent component of the HT response and is closely associated with multiple stress-responsive regulatory pathways.

Ferroptosis is marked by excessive LPO caused by disturbances in iron homeostasis and compromised antioxidant defenses (Xia et al. 2021; Gao et al. 2023). Accumulating evidence suggests that oxidative stress contributes to ferroptosis-related regulation under HT stress. Under HT stress, excessive ROS production promotes LPO, while iron dyshomeostasis expands the intracellular labile iron pool, thereby increasing ferroptotic vulnerability (Zhang et al. 2025). In response, fish may enhance glutathione peroxidase 4 (GPx4)/glutathione (GSH)-mediated antioxidant defenses and regulate iron transport and storage pathways to preserve redox and iron homeostasis during stress (Xia et al. 2021).

Bile acids (BAs) contribute importantly to the control of lipid metabolic balance. BAs participate in immune regulation, energy metabolism, and stress adaptation by activating transcription factors such as farnesoid X receptor (FXR), thereby exerting important effects on growth performance and disease resistance (Wang et al. 2023). Accordingly, BA homeostasis is closely associated with physiological health. Previous research has suggested that BA metabolism can undergo marked alterations in fish exposed to HT stress. For instance, HT stress has been reported to disturb BA homeostasis in *Lateolabrax maculatus*, accompanied by impaired hepatic FXR signaling, dysregulated BA synthesis and transport, and accumulation of hydrophobic BAs (Yang et al. 2026). Beyond its established metabolic functions, BA signaling is responsive to cellular stress and may participate in physiological processes involving redox homeostasis and ferroptosis-related changes (Wu et al. 2026; Tschuck et al. 2023; Liu et al. 2025, 2024a). However, the characteristics of BA metabolic responses to HT stress in *T. ovatus* remain insufficiently understood, limiting our understanding of hepatic homeostatic regulation under HT stress.

As a key metabolic and immunological hub in fish, the liver participates in detoxification, immune regulation, and stress responses, making it a major organ vulnerable to environmental challenges. The maintenance of hepatic functional homeostasis directly affects organismal physiological equilibrium. However, under HT stress, the processes underlying hepatic changes in *T. ovatus* remain incompletely resolved. Therefore, this study subjected *T. ovatus* to sustained HT stress over a 7-day period to systematically analyze changes in hepatic physiological homeostasis at multiple biological levels, including histological structure, oxidative stress, iron homeostasis, inflammation, immune response, and apoptosis. Furthermore, the reprogramming characteristics of the BA metabolic profile were systematically investigated, and the potential BA biomarkers were also identified. New insights from this study help clarify the hepatic responses of *T. ovatus* to HT stress and may support the formulation of targeted stress-mitigation approaches and functional feed additives.

## Results

### The survival of the fish

Both groups maintained a 100% survival rate during the 7-day experimental period. During routine observation, HT-exposed fish showed impaired schooling cohesion and decreased spontaneous swimming activity compared with control (CK) fish.

### Histological changes in the liver

**Fig. 1:**
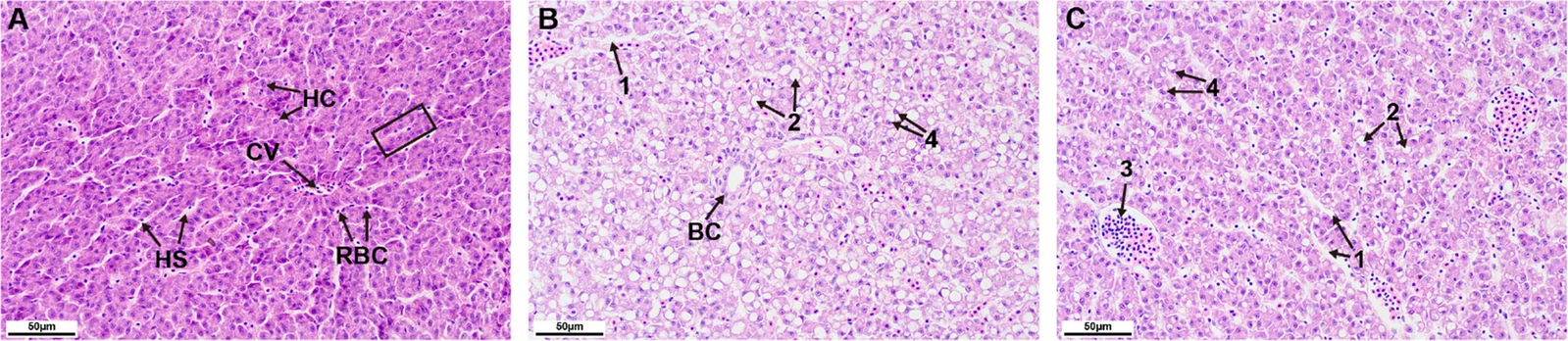
Histomorphological changes in the livers of *T. ovatus* after HT stress. **A** CK group. **B-C** HT group. H&E (400 ×): CV, central vein; HC, hepatocyte; HS, hepatic sinusoid; RBC, red blood cell; BC, bile canaliculus; boxed area, hepatic cord. 1, sinusoidal dilatation; 2, vacuolation; 3, inflammatory cell infiltration; 4, nuclear displacement

Liver sections from the CK group displayed normal histological architecture. Hepatocytes, the central vein, and hepatic sinusoids exhibited normal morphology, and hepatic cords were regularly organized around the central vein (Fig. 1A). Following HT stress, the liver exhibited pronounced histopathological alterations. Hepatocytes displayed disorganized arrangement, a loosened structure, and indistinct cellular boundaries, accompanied by marked cytoplasmic vacuolation. Some nuclei exhibited pyknosis, displacement, or even karyolysis. Sinusoidal enlargement and infiltration by inflammatory cells were additionally observed (Fig. 1B, C).

### The redox and stress responses in the liver

**Fig. 2:**
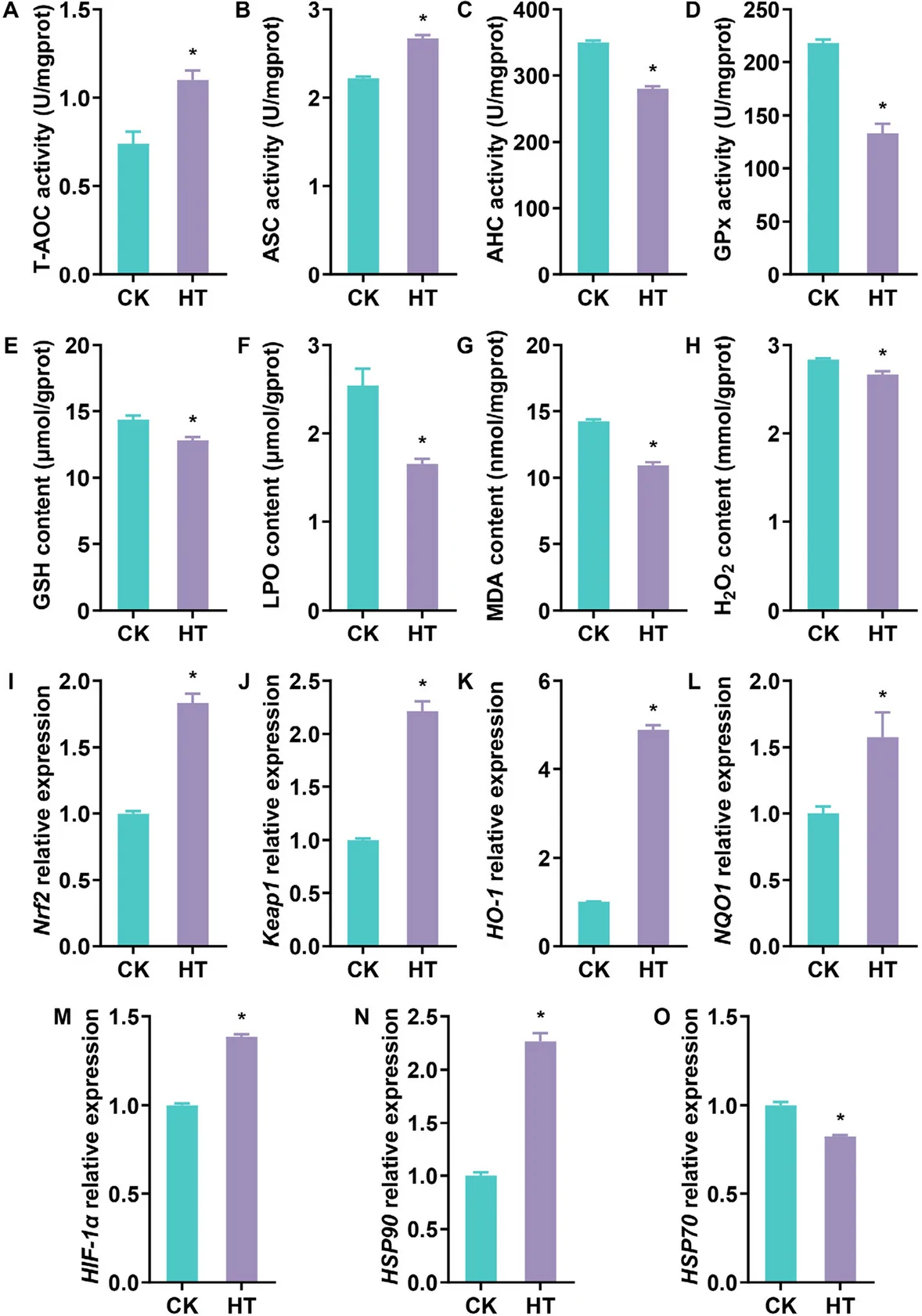
Changes in the redox and stress-related indexes in the liver of *T. ovatus* after HT stress. * indicates a significant difference between the two groups (*p* < 0.05)

Compared with the CK group, HT stress resulted in significantly higher activities of oxidative stress-associated biochemical indicators, including total antioxidant capacity (T-AOC) and anti-superoxide anion capacity (ASC). Conversely, parameters including anti-hydroxyl radical capacity (AHC), glutathione peroxidase (GPx), GSH, LPO, malondialdehyde (MDA), and hydrogen peroxide (H_2_O_2_) were all markedly reduced (*p* < 0.05) (Fig. 2A-H). HT stress significantly upregulated the expression of Nuclear factor erythroid 2-related factor 2 (Nrf2) pathway-related genes, including *Nrf2*, Kelch-like ECH-associated protein 1 (*Keap1*), heme oxygenase-1 (*HO-1*), and NAD(P)H quinone oxidoreductase 1 (*NQO1*) (*p* < 0.05) (Fig. 2I-L). Furthermore, HT stress altered the expression patterns of stress-responsive genes, with hypoxia-inducible factor 1-α (*HIF-1α*) and heat shock protein 90 (*HSP90*) expression being upregulated, whereas heat shock protein 70 (*HSP70*) expression was reduced (*p* < 0.05) (Fig. 2M-O).

### The ferroptotic responses in the liver

**Fig. 3:**
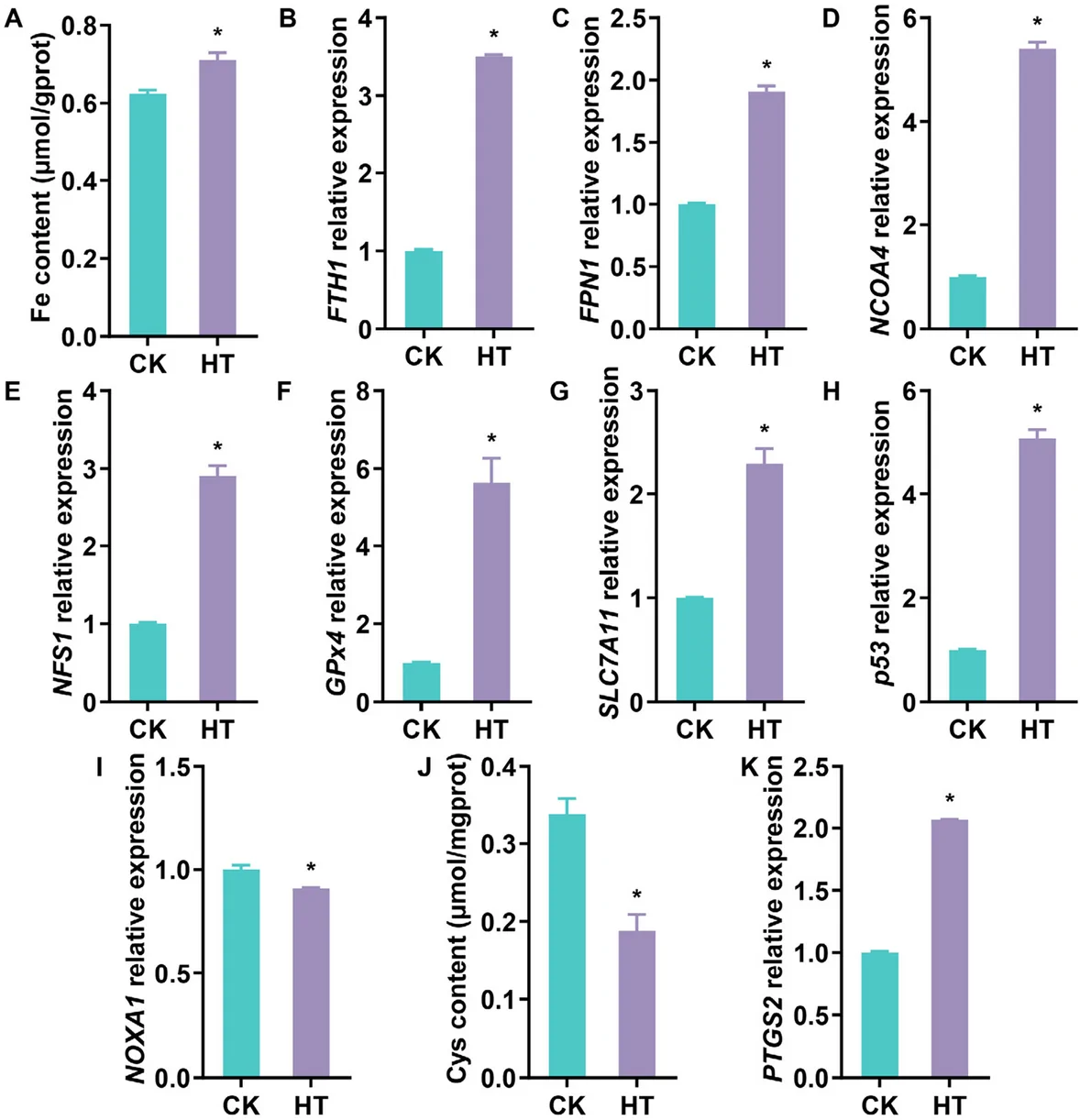
Changes in the ferroptosis-related indexes in the liver of *T. ovatus* after HT stress. * indicates a significant difference between the two groups (*p* < 0.05)

Relative to the CK group, HT stress resulted in elevated hepatic iron (Fe) levels and enhanced expression of ferritin heavy chain 1 (*FTH1*), ferroportin 1 (*FPN1*), nuclear receptor coactivator 4 (*NCOA4*), and cysteine desulfurase (*NFS1*) genes (*p* < 0.05) (Fig. 3A–E). In terms of the GPx4-mediated antioxidant pathway, *GPx4*, solute carrier family 7 member 11 (*SLC7A11*), and tumor protein p53 (*p53*) exhibited significant upregulation, whereas NADPH oxidase activator 1 (*NOXA1*) and cysteine (Cys) levels were markedly reduced (*p* < 0.05) (Fig. 3F–J). Furthermore, prostaglandin-endoperoxide synthase 2 (*PTGS2*), a ferroptosis-related marker gene, was significantly upregulated (*p* < 0.05) (Fig. 3K).

### Inflammatory and immune responses in the liver

**Fig. 4:**
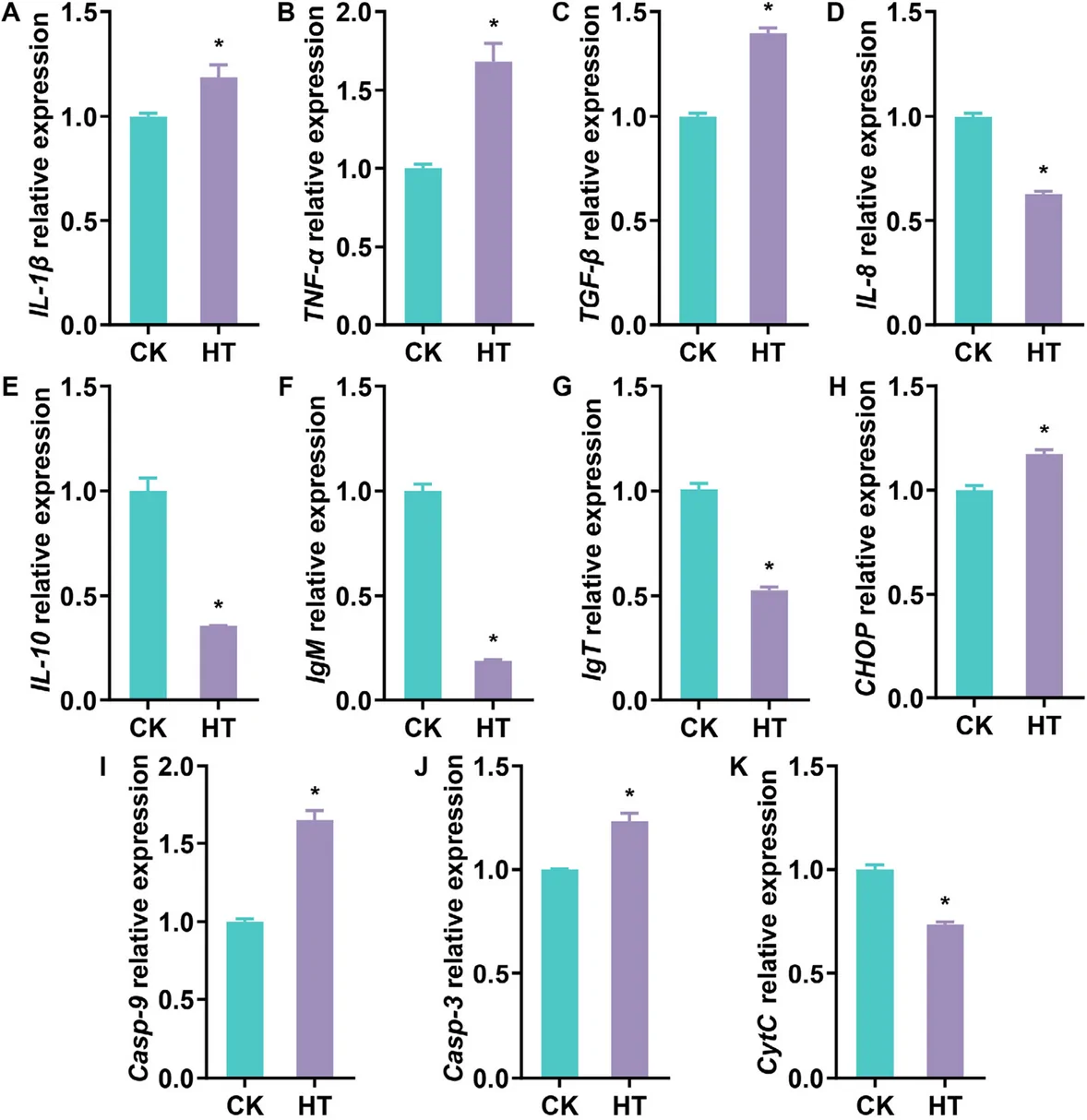
Changes in the inflammation-, immune-, and apoptosis-related indexes in the liver of *T. ovatus* after HT stress. * indicates a significant difference between the two groups (*p* < 0.05)

Relative to the CK group, HT stress markedly increased interleukin-1β (*IL-1β*), tumor necrosis factor-α (*TNF-α*), and transforming growth factor-β (*TGF-β*) expression, but decreased interleukin-8 (*IL-8*) and interleukin-10 (*IL-10*) expression (*p* < 0.05) (Fig. 4A-E). Additionally, HT stress markedly downregulated the immune-related genes immunoglobulin M (*IgM*) and immunoglobulin T (*IgT*) (*p* < 0.05) (Fig. 4F-G).

### The apoptotic responses in the liver

Relative to the CK group, HT stress induced significant increases in C/EBP homologous protein (*CHOP*), caspase-9 (*Casp-9*), and caspase-3 (*Casp-3*) expression, while reducing Cytochrome C (*CytC*) expression (*p* < 0.05) (Fig. 4H-K).

### Changes in the hepatic BA metabolic profiles

#### The basic overview of BA metabolites in the liver

**Fig. 5:**
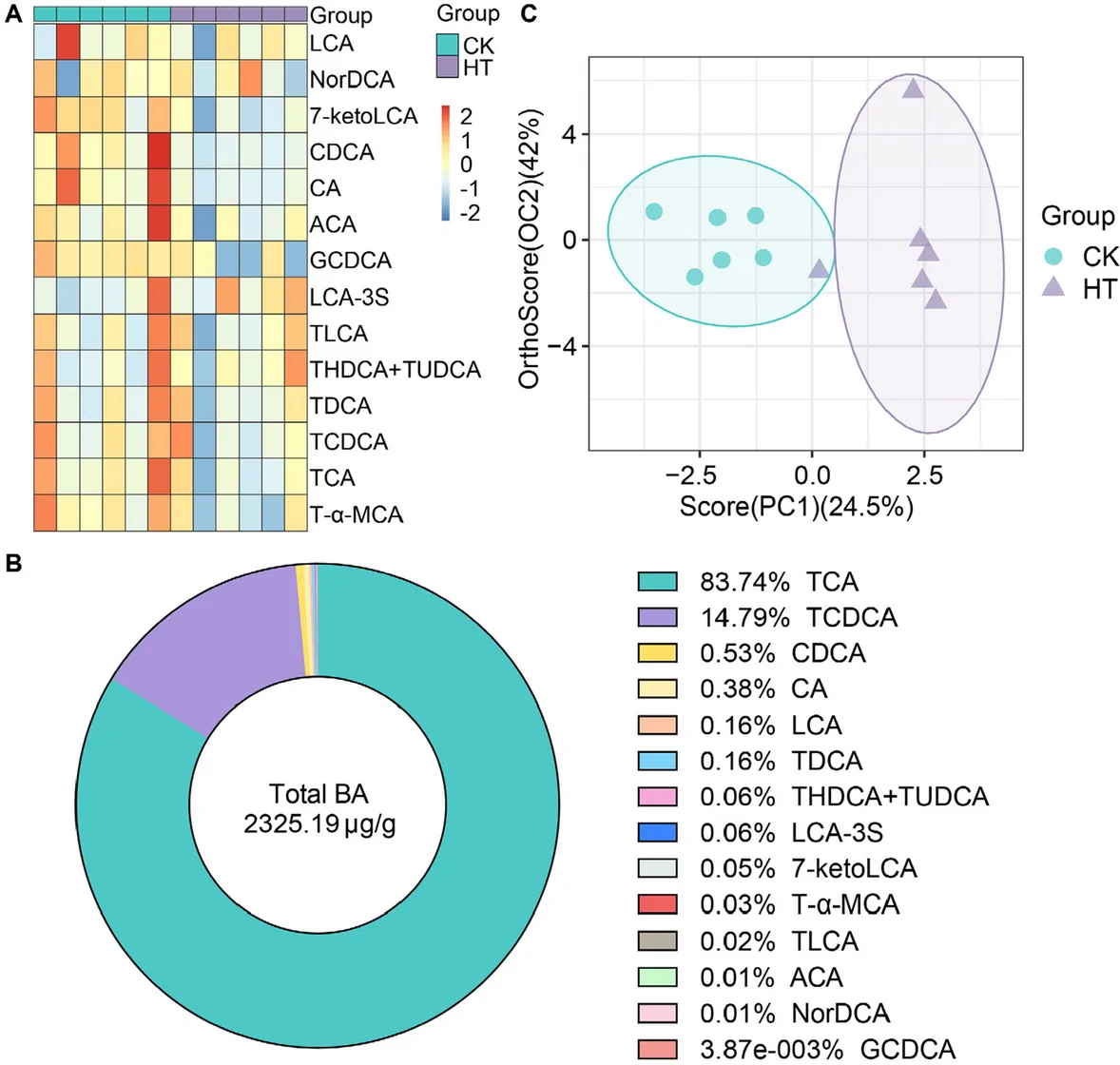
Changes in the BA profile in the liver of *T. ovatus* after HT stress. **A** The heatmap of the changes in 14 individual BA species. **B** BA composition. **C** The OPLS-DA plot based on BA profiles. LCA, lithocholic acid; NorDCA, 23-nordeoxycholic acid; 7-ketoLCA, 7-ketolithocholic acid; CDCA, chenodeoxycholic acid; CA, cholic acid; ACA, allocholic acid; GCDCA, glycochenodeoxycholic acid sodium salt; LCA-3S, lithocholic acid 3-sulfate sodium salt; TLCA, taurolithocholic acid sodium salt; THDCA + TUDCA, taurohyodeoxycholic acid sodium salt and tauroursodeoxycholic acid sodium salt; TDCA, taurodeoxycholic acid sodium salt; TCDCA, taurochenodeoxycholic acid; TCA, taurocholic acid sodium salt; T-α-MCA, tauro-α-muricholic acid sodium salt

Assessment of hepatic BA composition relied on liquid chromatography–tandem mass spectrometry (LC–MS/MS). 14 of the 39 targeted BA metabolites were detected (Fig. 5A). Among them, taurochenodeoxycholic acid (TCDCA) and taurocholic acid (TCA) accounted for the highest proportions, reaching more than 80% and 10%, respectively. These were followed by taurodeoxycholic acid sodium salt (TDCA), lithocholic acid (LCA), cholic acid (CA), and chenodeoxycholic acid (CDCA), each accounting for 0.1–1.0% (Fig. 5B). Among the HT and CK groups, an overall differentiation in metabolic profiles was demonstrated by the score plot derived from orthogonal partial least squares discriminant analysis (OPLS-DA) (Fig. 5C). However, one HT sample overlapped with the distribution of the CK group, indicating that individual variation existed within the HT group despite the overall separation between the two groups.

#### The variation characteristics of BA metabolites in the liver

**Fig. 6:**
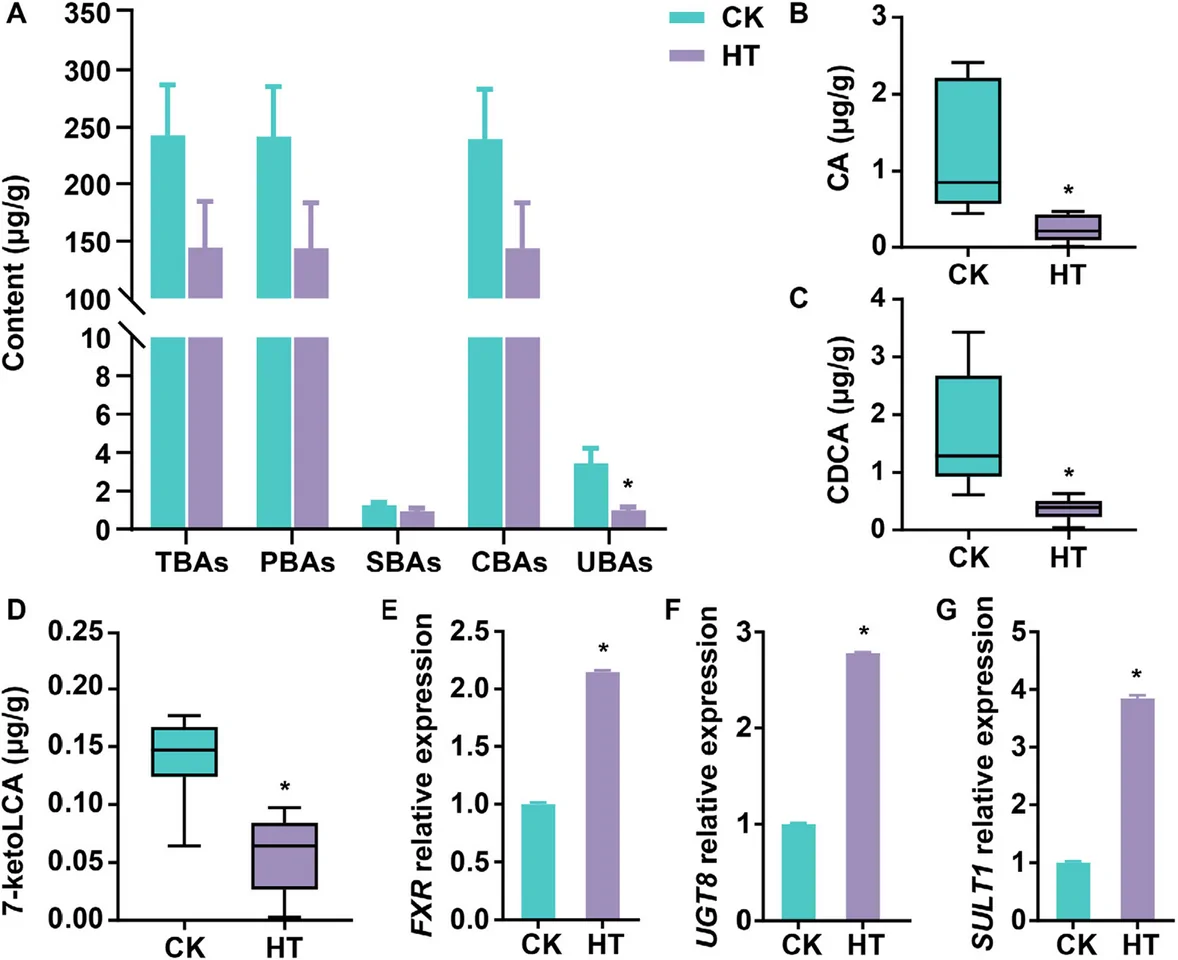
Changes in BA metabolites and related gene expression in the liver of *T. ovatus* after HT stress. **A** The levels of TBAs, PBAs, SBAs, CBAs and UBAs in the liver. **B-D** The significantly altered specific BA metabolites. **E–G** The expression of BA metabolism-related genes. * indicates a significant difference between the two groups (*p* < 0.05)

The changes in BA metabolites under HT stress were further analyzed. Primary bile acids (PBAs), secondary bile acids (SBAs), total bile acids (TBAs), and conjugated bile acids (CBAs) did not differ significantly (*p* > 0.05). In contrast, HT stress significantly decreased the abundance of unconjugated bile acids (UBAs) (*p* < 0.05) (Fig. 6A). Furthermore, individual BA profiling revealed that the levels of CA, CDCA, and 7-ketolithocholic acid (7-ketoLCA) were markedly decreased by HT stress (*p* < 0.05) (Fig. 6B-D).

#### The BA metabolism-related gene expression in the liver

Expression changes in genes involved in BA metabolism were then examined following HT stress. Relative to CK fish, HT stress significantly enhanced the expression of *FXR*, UDP-glucuronosyltransferase 8 (*UGT8*), and sulfotransferase 1 (*SULT1*) genes (*p* < 0.05) (Fig. 6E-G).

## Discussion

Frequent increases in water temperature during summer induce stress in *T. ovatus*, thereby increasing disease susceptibility and becoming a key constraint on healthy aquaculture. As a primary metabolic organ in teleosts, the liver is crucial for regulating stress adaptation, immune defense, and detoxification, and its functional integrity is essential for maintaining organismal homeostasis under HT stress. Nevertheless, the underlying hepatic mechanisms governing physiological adaptation to HT stress in *T. ovatus* have not been fully elucidated. As ectothermic organisms, fish exhibit an increase in metabolic rate with rising environmental temperature, which helps maintain energy homeostasis (Volkoff and Rønnestad 2020). However, HT stress may disrupt metabolic regulation and impair tissue homeostasis (Ern et al. 2023). In this study, no fish died throughout the 7-day HT stress, whereas marked hepatic histopathological alterations were observed in the HT group. These findings suggested that 7 days of exposure to 34 °C might not have been sufficient to reach a lethal threshold, but nevertheless imposed a significant sublethal challenge and disrupted hepatic homeostasis. On this basis, these changes were further interpreted across several biological dimensions.

Elevated temperature can induce cellular stress responses in fish. Heat shock proteins (HSPs) are key stress biomarkers induced under diverse stress conditions and are essential for protein folding and cellular homeostasis (Lee et al. 2023). In the current work, an enhanced expression of *HSP90* coupled with the suppression of *HSP70* was observed, suggesting differential regulation of HSP family members. Accumulating evidence indicates that HSP family members display distinct expression patterns depending on stress conditions and duration (Fan et al. 2021; Kaur et al. 2025; Sun et al. 2021). Therefore, the contrasting expression profiles of *HSP70* and *HSP90* might reflect their distinct functions or regulatory mechanisms under HT stress, possibly in association with alterations in molecular chaperone systems responsible for protein homeostasis. Hypoxia-inducible factors (HIFs) are key mediators of cellular responses (Wu et al. 2024). Our findings revealed increased *HIF-1α* expression, suggesting that HT stress might have elicited hypoxia-related signaling in the liver.

HT stress is well known to disturb cellular redox balance and trigger antioxidant defense responses in fish. The Keap1-Nrf2 signaling system induces the expression of redox-protective genes to facilitate ROS elimination and preserve redox homeostasis (Chen et al. 2020; He et al. 2020). The observed induction of *Nrf2*, *HO-1*, and *NQO1*, together with the rise in T-AOC and ASC activities, showed that hepatic antioxidant defenses were mobilized under HT stress. Such a compensatory response may have enhanced ROS scavenging capacity and effectively limited LPO. This interpretation is further supported by the significant declines observed in hepatic H_2_O_2_, LPO, and MDA. Although previous studies have generally reported increased LPO following HT stress (Liu et al. 2022a; Alfons et al. 2023), similar decreases in oxidative damage markers have also been reported in *Oncorhynchus mykiss* and *Clarias fuscus* under HT stress (Yousefi et al. 2022; Liu et al. 2024b). Accordingly, the reduced LPO may reflect an effective antioxidant response that limited excessive ROS accumulation and subsequent membrane lipid oxidation. Nevertheless, the decreases in AHC and GPx activities and GSH content suggested an imbalance in redox homeostasis despite the attenuation of LPO. Notably, *Keap1* mRNA was also upregulated together with *Nrf2*. Similar transcriptional patterns have likewise been observed in prior research (Liu et al. 2022b; Elbialy et al. 2024; Zeng et al. 2017; Janasik et al. 2018), which implied that *Keap1* transcription may also be dynamically regulated during oxidative stress. Therefore, the simultaneous induction of *Nrf2* and *Keap1* might reflect a complex transcriptional regulation of the Keap1–Nrf2 regulatory axis. Collectively, these findings suggested that despite enhanced antioxidant responses and diminished lipid oxidation products, redox homeostasis remained partially disrupted.

HT stress altered hepatic iron homeostasis and antioxidant defense, two processes closely associated with cellular susceptibility to ferroptosis. NCOA4 has been reported to promote ferritin degradation (Sun et al. 2025). The observed increase in hepatic Fe content and elevation of *NCOA4* expression suggested enhanced ferritin turnover, potentially contributing to an increase in intracellular bioavailable iron. Meanwhile, *FTH1* and *FPN1* can facilitate iron sequestration and export, whereas *NFS1* contributes to Fe–S cluster biosynthesis (Huang et al. 2025). Their coordinated upregulation therefore suggests adaptive regulation of iron storage, utilization, and transport in response to altered iron homeostasis under HT stress. Regarding antioxidant defense, GPX4-mediated detoxification of phospholipid peroxides represents a major defense mechanism against ferroptosis (Long et al. 2023a). Decreased *NOXA1* expression might reflect a lower ROS-generating potential, whereas the upregulation of *GPx4* suggested enhanced capacity for lipid peroxide detoxification.

In this study, the downregulation of *NOXA1* gene suggested reduced ROS-generating potential, whereas the upregulation of *GPx4* indicated enhanced capacity for lipid peroxide detoxification. This antioxidant process is commonly associated with SLC7A11-mediated cystine uptake and p53 regulation (Ma et al. 2021b; Fujii et al. 2023). Here, simultaneous increases in *SLC7A11* and *p53* expression suggested a compensatory regulation of the cystine/GSH axis under HT stress. However, intracellular Cys and GSH levels were decreased, which may reflect increased consumption during antioxidant responses. Notably, although the expression of the ferroptosis-associated marker *PTGS2* was significantly increased, hepatic LPO and MDA levels were significantly reduced, indicating that the excessive LPO characteristic of ferroptosis was not evident. Therefore, HT stress altered ferroptosis-related regulation in the liver, without evidence of ferroptosis execution, as characterized by coordinated changes in iron metabolism and adaptive antioxidant responses.

Redox imbalance may act as a signal regulating inflammatory and immune processes, and stress responses in aquatic animals are often accompanied by inflammatory activation and immune dysfunction (Faught and Schaaf 2024). HT stress also caused pronounced alterations in hepatic inflammatory and immune responses. Specifically, HT stress upregulated the expression of *IL-1β* along with *TNF-α*, suggesting enhanced hepatic pro-inflammatory responses. In contrast, anti-inflammatory genes did not exhibit a coordinated regulatory pattern. As an important anti-inflammatory cytokine, *TGF-β* suppresses pro-inflammatory signaling cascades (Zhang et al. 2022; Kuciński et al. 2025). Its expression was elevated under HT stress, while *IL-10* was significantly downregulated. This divergent regulatory pattern suggested that anti-inflammatory regulation might be dysregulated under HT stress. Such dysregulation was further reflected in immune cell recruitment and humoral immunity. The expression of *IL-8*, which promotes immune-cell recruitment to inflammatory sites (Klak et al. 2024), was downregulated. This decrease may indicate reduced recruitment of hepatic immune cells despite heightened inflammatory signaling. Similarly, the expression of *IgM* and *IgT* was also reduced. Because these immunoglobulins mediate systemic and mucosal immunity (Hansen et al. 2005; Xu et al. 2024), this reduction suggests suppressed humoral immune function and potentially increased susceptibility to pathogen infection. Collectively, these findings suggested that HT stress disturbed hepatic immune homeostasis by activating the pro-inflammatory response, disturbing anti-inflammatory regulation and immune cell recruitment, and suppressing humoral immune function.

Persistent stress and inflammatory responses may alter apoptosis-related regulatory processes (Yan et al. 2021). CHOP functions as a pivotal transcriptional regulator within the apoptotic cascade triggered by endoplasmic reticulum (ER) stress (Xu et al. 2021). Casp-9 and Casp-3 are key caspases involved in apoptotic signal transduction (Cheng et al. 2018). Here, following HT stress, the expression of *CHOP*, *Casp-9*, and *Casp-3* genes was upregulated, whereas *CytC* expression was downregulated in the liver. These findings indicated that HT stress triggered transcriptional responses in apoptosis-related genes and might have affected the functional homeostasis of apoptosis.

BA metabolism participates in the stress response of fish by modulating hepatic lipid homeostasis, immune regulation, and antioxidant defense, and its dysregulation may contribute to metabolic disturbances (Wang et al. 2023). In the current work, HT stress significantly lowered hepatic UBAs together with several individual BAs, including CA, CDCA, and 7-ketoLCA. These alterations in BA composition suggested selective remodeling of the hepatic BA pool, particularly within the UBA fraction. Because excessive accumulation of hydrophobic UBAs may impair membrane integrity and promote oxidative injury (Li et al. 2022), the reduction in UBAs might represent an adaptive response that limits BA-associated cytotoxicity during HT stress. Consistent with these metabolic changes, HT stress significantly upregulated *FXR*, *UGT8*, and *SULT1*, indicating enhanced regulation of BA homeostasis. FXR is a key regulator of BA homeostasis (Perino and Schoonjans 2022), whereas UGT- and SULT-related enzymes participate in the detoxification and elimination of hydrophobic BAs (Kastrinou Lampou et al. 2023; Meech et al. 2015). The parallel induction of these genes might constitute a compensatory adjustment that supported BA balance.

Notably, recent studies have suggested that FXR-mediated BA signaling contributes to oxidative stress adaptation and the control of lipid oxidation. Activation of FXR has been reported to modulate the GSH–GPX4 axis and limit LPO (Tschuck et al. 2023; Liu et al. 2025). In the current work, *FXR* upregulation occurred concurrently with increased *GPx4* expression and reduced LPO and MDA levels. Although no direct mechanistic relationship can be established, these findings suggested that BA metabolic remodeling might occur in parallel with oxidative stress adaptation and lipid peroxide defense during HT stress.

Several limitations should nevertheless be considered. Although the sample size was sufficient to support the validity of the present conclusions, it may still have constrained the sensitivity of the statistical analyses to some extent. Second, feeding behavior and growth performance were not quantitatively monitored, limiting our ability to link the molecular findings to overall physiological responses. In addition, several biochemical indicators of hepatic injury and oxidative stress, including alanine aminotransferase (ALT) and hepatic ROS levels, were not measured. Future studies incorporating these indicators with larger sample sizes could offer deeper insights into the stress response elicited by elevated temperature.

## Conclusions

**Fig. 7:**
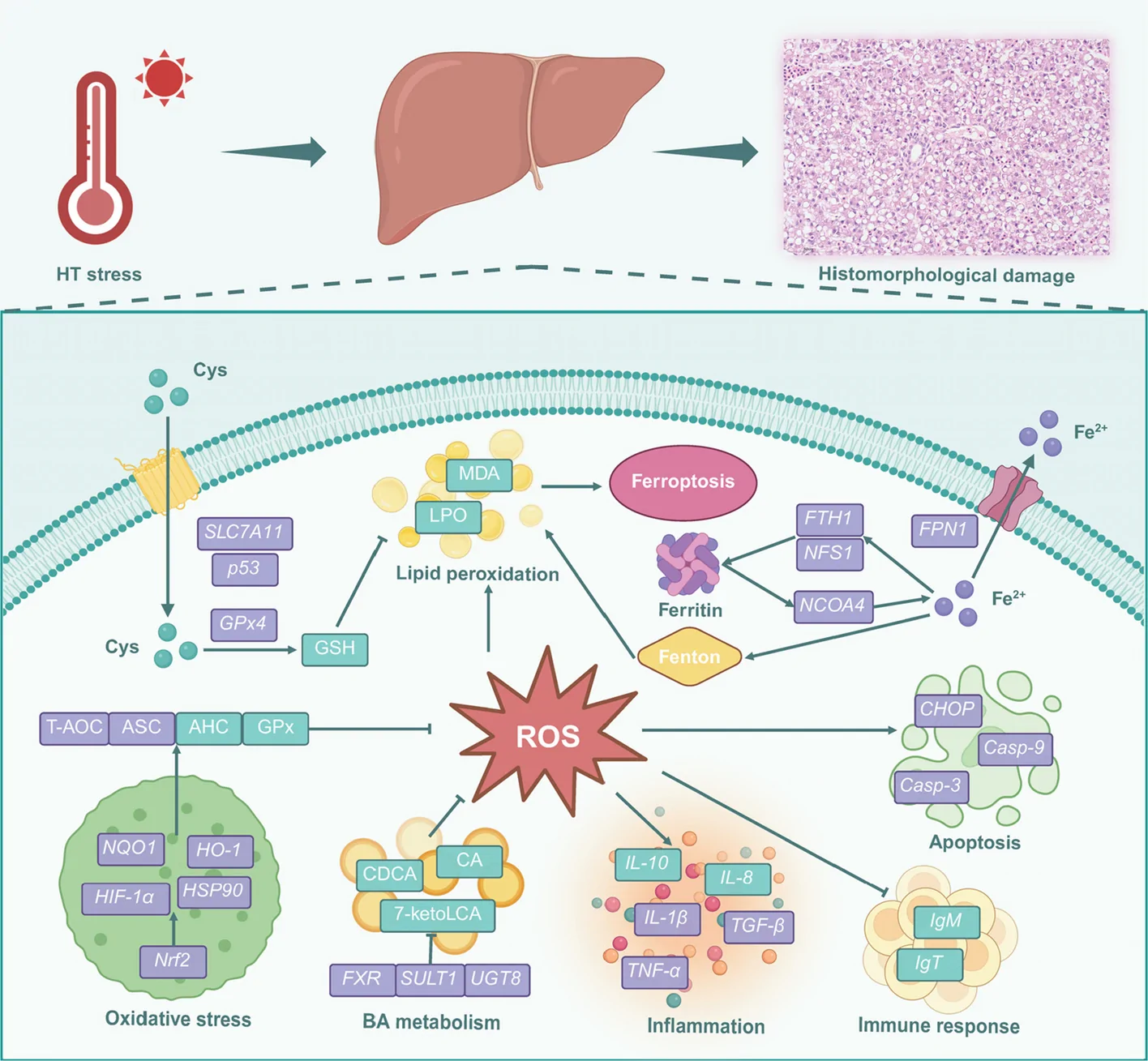
A mechanistic deduction of the physiological responses in the liver of *T. ovatus* to HT stress. Text in purple represents increased indicators, while text in green represents decreased indicators

Our findings indicated that exposure to HT stress impaired hepatic homeostasis in *T. ovatus*. Specifically, HT challenge induced histopathological alterations and disturbed hepatic redox balance. Induction of Nrf2 signaling may reflect an antioxidant compensation to HT stress. Redox perturbation might further affect GSH metabolism, iron storage and iron transport. These changes were accompanied by inflammatory responses, downregulation of immunoglobulin genes, and apoptosis-related transcriptional responses. Furthermore, HT stress altered hepatic BA metabolic homeostasis, characterized by reductions in the BA pool and free BA levels together with modulation of FXR-related detoxification and excretion responses, which may influence BA transformation and clearance (Fig. 7). Collectively, these findings advance our understanding of hepatic responses to HT stress in *T. ovatus* and may inform the formulation of BA-based functional feed supplements for alleviating HT-induced stress.

## Materials and methods

### Fish rearing conditions

Considering the importance of the juvenile stage in *T. ovatus* aquaculture and the vulnerability of juveniles to changing ambient conditions (Han et al. 2020; Li et al. 2023b), juvenile *T. ovatus* (mean body weight 9.0 ± 1.6 g) were selected to evaluate their physiological responses to HT stress. Before HT exposure, fish completed a 3-day conditioning period in 300 L culture vessels stocked with 20 individuals each. Filtered natural seawater was used for culture, with salinity maintained at 30.0‰, pH at 7.9–8.0, and temperature at 29.0 ± 0.2 °C. Fish were given given a commercially formulated diet (Gongli Formula Feed for Pampus Fish, Guangdong Yuequn Ocean Biotechnology Co., Ltd., Guangdong, China) to apparent satiation at two feeding times per day, with uneaten feed and fecal matter regularly cleared from the tanks. Table S1 summarizes the manufacturer's proximate composition data for the commercial diet.

### HT stress treatment and sample collection

Following the 3-day acclimation period, 120 fish were allocated at random to either the CK treatment or the HT treatment. For every treatment, 3 circular tanks served as replicates, with 20 fish stocked in every tank. The CK treatment remained at 29.0 ± 0.2 °C. For HT exposure, water started at the same temperature and was raised by 1 °C each hour until 34.0 °C was reached, after which it was kept constant. The rearing water temperature in Guangdong during summer frequently reaches as high as 34 °C, with earlier studies documenting physiological stress responses in *T. ovatus* exposed to 34 °C (Li et al. 2023a; Han et al. 2020). Therefore, 34 °C was used as the HT exposure temperature in the present study. Fresh seawater adjusted to the respective treatment temperature was used for daily water renewal during the stress experiment. All culture variables other than temperature were matched between CK and HT treatments. Mortality and overt behavioral changes were monitored throughout the experimental period. HT exposure lasted for 7 days, a duration commonly used in studies of various fish species (Han et al. 2020; Hu et al. 2023).

After the exposure ended, 18 fish were selected through random sampling from every tank. For histological analysis, liver samples obtained from three fish per tank were preserved using 4% paraformaldehyde. For biochemical assays, liver samples were obtained from six fish per tank and preserved. Liver samples from three fish were collected for subsequent gene expression measurements and preserved in RNAFollow solution. Liver tissues from the three fish within the same tank were combined prior to RNA extraction, with each tank serving as one biological replicate (*n* = 3 tanks per group) for each treatment group. 6 fish provided liver tissues for subsequent BA metabolomics.

### Histomorphological analysis

Following fixation in 4% paraformaldehyde, liver tissues underwent graded dehydration, clarification with xylene, and subsequent paraffin infiltration. Using a microtome (Leica RM2016, Wetzlar, Germany), the resulting tissue specimens were cut into 4-μm sections. For each fish, three non-consecutive liver tissue slices were selected and stained with hematoxylin and eosin. Five randomly selected microscopic fields from each section were assessed and imaged.

### Biochemical analysis

Liver samples were homogenized in 0.9% normal saline for the preparation of 10% tissue suspensions. After centrifugation (3500 rpm, 4 °C, 15 min), the resulting supernatant was used for biochemical measurements. Commercial kits were used for the determination of T-AOC, AHC, ASC, and GPx activities together with H_2_O_2_, LPO, MDA, GSH, Fe, and Cys concentrations. A microplate reader (Tecan, Grödig, Austria) was used to obtain all biochemical measurements.

### Gene expression analysis

Hepatic total RNA was obtained using TRIzol (Invitrogen, Carlsbad, USA). RNA quantity and purity were evaluated, followed by reverse transcription for cDNA preparation. Target transcript levels were quantified using quantitative real-time PCR (qPCR). Table S2 contains the qPCR primer sequences. Expression data were normalized against *β-actin*. Primer amplification efficiencies were evaluated using standard curves and melting curve analysis. qPCR reactions and cycling conditions are detailed in the Supplementary Materials. Following Livak and Schmittgen (2001), relative gene expression levels were determined.

### The metabolomic profiling of BAs

Liver tissues were processed by methanol extraction. BAs in the liver samples were characterized and quantified using an LC–MS/MS approach. Quality control analyses were conducted throughout mass spectrometric data acquisition. Metabolic separation between CK and HT treatments was characterized through OPLS-DA. False discovery rate (FDR) was adjusted according to the Benjamini–Hochberg (BH) method. BA metabolites having *p* < 0.05 were considered significantly different. R software (v4.0.3) was used to conduct hierarchical cluster analysis.

### Statistical analysis

Measurements from fish sharing a tank were consolidated into a single mean value, and each tank was considered one replicate. Normality and variance homogeneity were assessed for all the datasets. Between-group comparisons were carried out in SPSS 27.0 using independent-samples *t*-test. Values are summarized as mean ± standard error of the mean (SEM), and *p* values below 0.05 were taken to denote statistical significance.

Further methodological information is available in the Supplementary Materials.

## Supplementary Information

Supplementary Material 1: Table S1 Proximate composition of the commercial diet. Table S2 The primer sequences. Detailed materials and methods.

## Data Availability

Not applicable.

## Abbreviations

7-ketoLCA: 7-Ketolithocholic acid
AHC: Anti-hydroxyl radical capacity
BA: Bile acid
CDCA: Chenodeoxycholic acid
CA: Cholic acid
CHOP: C/EBP homologous protein
CK: Control
CytC: Cytochrome C
Casp-3: Caspase-3
Casp-9: Caspase-9
Cys: Cysteine
Fe: Iron
FPN1: Ferroportin 1
FXR: Farnesoid X receptor
FTH1: Ferritin heavy chain 1
GPx: Glutathione peroxidase
GSH: Glutathione
GPx4: Glutathione peroxidase 4
HIF-1α: Hypoxia-inducible factor 1-α
HO-1: Heme oxygenase-1
HSP90: Heat shock protein 90
HSP70: Heat shock protein 70
HT: High-temperature
H₂O₂: Hydrogen peroxide
IgT: Immunoglobulin T
IgM: Immunoglobulin M
IL-10: Interleukin-10
IL-8: Interleukin-8
IL-1β: Interleukin-1β
Keap1: Kelch-like ECH-associated protein 1
LPO: Lipid peroxidation
MDA: Malondialdehyde
NCOA4: Nuclear receptor coactivator 4
NFS1: Cysteine desulfurase
NQO1: NAD(P)H quinone oxidoreductase 1
NOXA1: NADPH oxidase activator 1
Nrf2: Nuclear factor erythroid 2-related factor 2
p53: Tumor protein p53
PTGS2: Prostaglandin-endoperoxide synthase 2
ROS: Reactive oxygen species
SLC7A11: Solute carrier family 7 member 11
SULT1: Sulfotransferase 1
T-AOC: Total antioxidant capacity
TGF-β: Transforming growth factor-β
TNF-α: Tumor necrosis factor-α
UGT8: UDP-glucuronosyltransferase 8
