# Supplementary material for: High-temperature stress response in the liver of Trachinotus ovatus: a cascade of oxidative stress, immunity, and bile acid metabolic reprogramming
Source: Stress Biol. 2026 Oct 19;6(1):71. doi: 10.1007/s44154-026-00344-y (PMC13680597; doi:10.1007/s44154-026-00344-y)
Supplement: Supplementary file 1 [file 44154_2026_344_MOESM1_ESM.docx]

**Table S1** Proximate composition of the commercial diet used in this study.

| Component | Content (%) |
| --- | --- |
| Crude protein | ≥46.0 |
| Crude lipid | ≥6.0 |
| Crude fiber | ≤5.0 |
| Crude ash | ≤18.0 |
| Lysine | ≥2.1 |
| Total phosphorus | ≥1.2 |
| Moisture | ≤12.0 |

Table S2 The primer sequences used in this study

| Gene | Forward primer (5'−3') | Reverse primer (5'−3') |
| --- | --- | --- |
| *Nrf2* | AGCTTGGCCTTCATCAAAT | GAGTATGGCTGTCCTTCTTCA |
| *Keap1* | AGAGGATGGAGATGGCACAG | CATTGGTTGGTCTTGGGATT |
| *NQO1* | TGGTCCAGGTGTCACGTCTTCC | GACTTGGCGGTGTAGTGCTTGG |
| *HO-1* | AGAAGATTCAGACAGCAGCAGAACAG | TCATACAGCGAGCACAGGAGGAG |
| *NCOA4* | AAGATCGACGAATCAGAGTTCACCATC | GTCTGACACAGTCTCCATCGCATC |
| *PTGS2* | TGTCCCGTGGCGTCCTTCC | CCTGAGTGAGACGTGCTTGAGTTG |
| *NOXA1* | GCGTGCCAGCTCAACTCAGG | AGGGACAGACAGAGCCACAGTG |
| *p53* | ACAACCACTCAGATCAATCGCTTCC | GGAGCCAAGACAATGACAGCCTAC |
| *FTH1* | CCGCCGCCGCAAGTGTG | TGACAGACCCAGGACTATTGTGAGG |
| *GPx4* | GACAGATATTGATGGCAACGTGGTTTC | GCTGCTGGTGGACTTGTGATGG |
| *SLC7A11* | GCTAGACCACTACAGTCCATCACAAC | TGCTGCAACACTTCCATCACTCTG |
| *NFS1* | ACCTCCCAGTCCAGCAGAACG | TGTGTGACCTGTGTGTAACTGTGTG |
| *FPN1* | CGTCCAGAACAGTTGCGTCATC | AGTTGTCAGAATCCATCCATTGTAGAG |
| *Casp-9* | GAATGGCGTCCGTCTGGTCATC | GGCAGCACGTCTCAGTTCAGC |
| *Casp-3* | GCTGCTCTACTGCTTCTGCTGATG | TGGCTGAGGATTGTGATGTTGCTG |
| *CHOP* | CGGAGTTTCTGGATGTTTTGGA | AGGAGGAGGAAGAGGAGGATGA |
| *CytC* | AACCACTTCTACCACCACCATAC | TTCTATACGACACAGTAGCAACAAAC |
| *FXR* | CATGCTGGACGTGCTGAGGAAG | TCTGCGTGGTAATGGTTGAGTGTTC |
| *SULT1* | TACATTCACAAGTTCATGCGAGGAGAG | TGCAAATAGAGACCAGTGGGATTCAAG |
| *UGT8* | AACACAGGAAAGACACATTGGAGGAC | AGGAAGGCAGGAAGACGACAGAG |
| *HIF-1α* | TCAGCACAGGACAGTACAGGATG | TGTTGTTATAGATGACAGTGGCTTGG |
| *HSP90* | GTGGTGTGGTGGACTCAGAGG | TTCTTGACAATGTTCTTGCGAATGAC |
| *HSP70* | AATTCTCCATCACAGACATCGTTCC | TCGCAATCACTCAGTCCTTCCTC |
| *IgT* | TGCCAGTGGGACCAGCCT | TGGAAGATCCGCCATGAGTTACT |
| *IgM* | TGCTGGCACCCCTTTTACAT | CCTCCCAAAAAATTCTCTCAACAG |
| *TNF-α* | CGCAATCGTAAAGAGTCCCA | AAGTCACAGTCGGCGAAATG |
| *IL-1β* | GCGATTTGGTGCGATTTCT | CTCTACTGGCTTGTTGTCTTGC |
| *IL-8* | GAGAAGCCTGGGAATGGA | GAGCCTCAGGGTCTAAGCA |
| *IL-10* | CGTCCTGGCTCTCTTGTCTCTCCTC | TGTCCATGTCATTGTTTGCCTCATA |
| *TGF-β* | TATCCCTCTACAACAGCACCA | GGTCAGCAGGCGGTAATC |
| *β-actin* | TGAACCCCAAAGCCAACAGG | CCGCAGGACTCCATACCAAG |

**Materials and Methods**

**Fish rearing conditions**

Considering the importance of the juvenile stage in *T. ovatus* aquaculture and its sensitivity to environmental fluctuations (Han et al. 2020; Li et al. 2023b), juvenile *T. ovatus* (mean body weight 9.0 ± 1.6 g) were selected to evaluate their physiological responses to HT stress and were obtained from the Shenzhen Base of the South China Sea Fisheries Research Institute. Prior to the HT stress experiment, fish were acclimated for 3 days in 300 L tanks at a stocking density of 20 fish per tank under continuous aeration and flow-through conditions. The rearing water consisted of filtered natural seawater maintained at a salinity of 30.0‰, a pH of 7.9–8.0, and a temperature of 29.0 ± 0.2 °C. During acclimation, nitrite and ammonia nitrogen concentrations remained below 1.0 mg/L and 0.5 mg/L, respectively, and dissolved oxygen was maintained above 6.0 mg/L. Fish were fed a commercial diet (Gongli Formula Feed for Pampus Fish, Guangdong Yuequn Ocean Biotechnology Co., Ltd., Guangdong, China) to apparent satiation twice daily, and residual feed and feces were removed promptly. The manufacturer's proximate composition of the commercial diet is presented in Table S1.

**HT stress treatment and sample collection**

Following the 3-day acclimation period, 120 fish were randomly divided into two experimental groups: the CK group and the HT group. Each group contained three circular tanks (inner diameter 98.0 cm, depth 100.0 cm, effective water volume 300 L), with 20 fish per tank. The temperature treatments consisted of two regimens: the CK group was maintained at 29.0 ± 0.2 °C, whereas the HT group was maintained at 34 °C that was subjected to a gradual increase from 29.0 ± 0.2 °C to 34.0 °C at a rate of approximately 1 °C per hour. The rearing water temperature in *T. ovatus* in Guangdong during summer frequently reaches as high as 34 °C, and previous studies have also demonstrated that this temperature can trigger stress responses in *T. ovatus* (Li et al. 2023a; Han et al. 2020). Therefore, 34 °C was selected as the exposure temperature for HT stress in the present study. During the stress experiment, the rearing water was renewed daily with fresh seawater pre-adjusted to the corresponding temperature. Except for water temperature, all other culture conditions, including water quality parameters, were kept consistent between the two groups and with those during the acclimation period. Mortality and overt behavioral changes were monitored throughout the experimental period. To investigate the effects of chronic HT stress on the physiological homeostasis of *T. ovatus*, the HT exposure in the present study lasted for 7 days, which is also a duration widely used in *T. ovatus* and other marine fish (Han et al. 2020; Hu et al. 2023).

At the end of the stress experiment, 18 fish were randomly selected from each tank and anesthetized with 10.0 mg/L eugenol. Liver tissues were collected immediately after dissection. For each tank, liver samples from 3 fish were fixed in 4% paraformaldehyde and stored at 4 °C for histological analysis. Liver samples from 6 fish were stored at −80 °C for biochemical analysis. For gene expression analysis, the liver samples from 3 fish were immersed in RNAFollow solution, kept at 4 °C for 24 h, and then stored at −80 °C. Liver tissues from the three fish within the same tank were pooled prior to RNA extraction, resulting in three biological replicates (n = 3 tanks per group) for each treatment group. Liver samples from 6 fish were stored at −80 °C for BA metabolomic analysis.

**Histomorphological analysis**

The livers were fixed in 4% paraformaldehyde for 24 h, and then rinsed under running water for 30 min. The samples were dehydrated through a graded ethanol series (70%, 80%, 90%, and 100%), cleared in xylene, and embedded in paraffin. Subsequently, 4 μm sections were prepared from the paraffin blocks using a microtome (Leica RM2016, Wetzlar, Germany). For each fish, three non-consecutive liver sections were prepared and subjected to hematoxylin and eosin (H&E) staining. Five randomly selected microscopic fields from each section were examined and imaged using a light microscope (Olympus, Tokyo, Japan).

**Biochemical analysis**

Liver samples were homogenized in 0.9% normal saline for the preparation of 10% tissue suspensions. The supernatant was collected following centrifugation (3500 rpm, 15 min, 4 °C) for biochemical measurements. The activities of T-AOC, AHC, ASC, and GPx, as well as the contents of H_2_O_2_, LPO, MDA, GSH, Fe, and Cys, were measured. All assays were performed using commercial kits (Nanjing Jiancheng Bioengineering Institute, Nanjing, China). Total protein content was measured using the Coomassie brilliant blue method. All measurements were performed using a microplate reader (Spark, Tecan, Grödig, Austria).

**Gene expression analysis**

Total RNA was isolated from livers using TRIzol reagent (Invitrogen, Carlsbad, CA, USA), followed by DNase I treatment to remove genomic DNA. The RNA quality and concentration were assessed by 1.0% agarose gel electrophoresis and using a NanoDrop 2000 spectrophotometer (Thermo Fisher Scientific, USA), respectively. cDNA was synthesized using the SweScript RT II First Strand cDNA Synthesis Kit (Servicebio, Wuhan, China).

The relative expression levels of target genes were determined by quantitative real-time PCR (qPCR) using the SGExcel Fast SYBR qPCR Mixture Kit (Sangon Biotech, Shanghai, China) on a real-time PCR system (CG-02, Heal Force, Shanghai, China). The specific primer sequences are provided in Table S2, and all primers were synthesized by Sangon Biotech (Shanghai, China). *β-actin* served as the reference gene for normalization. Primer amplification efficiencies were evaluated using standard curves and melting curve analysis. Each qPCR reaction (15 μL) contained 7.5 μL of 2× SGExcel Fast SYBR Mixture, 0.4 μL of each forward and reverse primer (10 μmol/L), 1.2 μL of cDNA template, and 5.5 μL of RNase-free water. The qPCR cycling conditions were as follows: 95 °C for 3 min, followed by 40 cycles of 95 °C for 5 s and 60 °C for 20 s. Gene expression was calculated based on the method proposed by Livak and Schmittgen (Livak and Schmittgen 2001).

**The metabolomic profiling of BAs**

Six liver samples from each group were used for BA metabolomic analysis. After pretreatment, the liver samples were extracted with methanol and centrifuged to obtain the supernatants. BAs in the liver samples were characterized and quantified using an LC–MS/MS approach. Liquid chromatographic separation was performed using an EXion LC liquid chromatography system (AB SCIEX, USA) equipped with an ACQUITY UPLC^®^ BEH C18 column (2.1 × 100 mm, 1.7 μm; Waters, USA). Mass spectrometric detection was performed with an AB6500+ tandem mass spectrometer (AB SCIEX, USA). Data acquisition was conducted in multiple reaction monitoring (MRM) mode, and quality control analyses were performed. OPLS-DA was performed to evaluate metabolic differences between the HT and CK groups. Differential BA metabolites were identified based on *p* < 0.05. The false discovery rate (FDR) was controlled using the Benjamini–Hochberg (BH) procedure. Hierarchical clustering analysis was performed using R software (v4.0.3).

**Statistical analysis**

Each tank was treated as an experimental unit for statistical analysis. Values from individual fish within the same tank were averaged to obtain a single value per tank. Normality and variance homogeneity were assessed for all the datasets. Statistical analyses were performed using SPSS 27.0 software with an independent-samples *t*-test. Data were expressed as mean ± standard error of the mean (SEM), with statistical significance defined at *p* < 0.05.
